# Fifth-Generation (5G) mmWave Spatial Channel Characterization for Urban Environments’ System Analysis

**DOI:** 10.3390/s20185360

**Published:** 2020-09-18

**Authors:** Leyre Azpilicueta, Peio Lopez-Iturri, Jaime Zuñiga-Mejia, Mikel Celaya-Echarri, Fidel Alejandro Rodríguez-Corbo, Cesar Vargas-Rosales, Erik Aguirre, David G. Michelson, Francisco Falcone

**Affiliations:** 1 School of Engineering and Sciences, Tecnologico de Monterrey, Monterrey 64849, NL, Mexico; jaime.zuniga@tec.mx (J.Z.-M.); mikelcelaya@gmail.com (M.C.-E.); alek.bsb@gmail.com (F.A.R.-C.); cvargas@tec.mx (C.V.-R.); 2 Electric, Electronic and Communication Engineering Department, Public University of Navarre, 31006 Pamplona, Navarra, Spain; peio.lopez@unavarra.es (P.L.-I.); erik.aguirre@unavarra.es (E.A.); francisco.falcone@unavarra.es (F.F.); 3 Institute of Smart Cities, Public University of Navarre, 31006 Pamplona, Navarra, Spain; 4 Electrical and Computer Engineering Department, University of British Columbia, Vancouver, BC V6T 1Z4, Canada; davem@ece.ubc.ca

**Keywords:** mmWave channel propagation, 5G, 3D Ray Launching, channel modeling

## Abstract

In this work, the channel characterization in terms of large-scale propagation, small-scale propagation, statistical and interference analysis of Fifth-Generation (5G) Millimeter Wave (mmWave) bands for wireless networks for 28, 30 and 60 GHz is presented in both an outdoor urban complex scenario and an indoor scenario, in order to consider a multi-functional, large node-density 5G network operation. An in-house deterministic Three-Dimensional Ray-Launching (3D-RL) code has been used for that purpose, considering all the material properties of the obstacles within the scenario at the frequency under analysis, with the aid of purpose-specific implemented mmWave simulation modules. Different beamforming radiation patterns of the transmitter antenna have been considered, emulating a 5G system operation. Spatial interference analysis as well as time domain characteristics have been retrieved as a function of node location and configuration.

## 1. Introduction

The path towards the progressive adoption of context-aware environments within the framework of smart cities and smart regions, is one of the main drivers for the current development and future deployment of multiple wireless communication systems within a HetNet architecture [1]. A large number of devices will potentially require communication capabilities within the development of the Future Internet (FI) and the Internet of Things (IoT), demanding high levels of connectivity, larger bandwidth requirements and lower delays, among others. In this sense, Fifth-Generation (5G) wireless networks rely on the use of spectrum blocks above 6 GHz, within the Millimeter Wave (mmWave) range, in order to increase capacity as well as to reduce overall interference levels of the heavily loaded sub-6 GHz frequency bands. Among the proposed requirements within 5G, we have an achievable Round-Trip Time (RTT) delay on the order of 1 ms, as compared to Long-Term Evolution (LTE) or Wireless Local Area Networks (WLAN) RTT values in excess of 20 ms, and the Device-to-Device (D2D) and Machine-Type Communications (MTC). Such attributes enable the provision of services in multiple application areas, such as manufacturing/Industry 4.0, healthcare/smart health, smart grids, autonomous vehicles/Intelligent Transportation Systems (ITS) or entertainment, among others [1,2]. These new services in turn impose additional demands in terms of network architecture, such as precise control of traffic imbalance, which requires strict interference control, aided by strategies such as phantom cells or uplink/downlink decoupling. Moreover, the advent of IoT enabled scenarios conveys a substantial increase in transceiver density, stressing the need to further control overall interference levels. Therefore, wireless channel characterization in terms of received power levels, intra-system and inter-system interference and subsequent Quality of Service (QoS) metric estimation is compulsory. 

Adequate network deployment strategies as well as subsequent system level optimization requires wireless channel characterization and radio planning, in order to estimate coverage/capacity relations, strongly related to network topology, traffic characterization and the surrounding environment. Network use cases are evolving towards context-aware scenarios, created by smart cities and smart regions, in which different systems (such as vehicular networks/ITS, MTC or ambient assisted living, among others) exhibit dynamic QoS thresholds. In the case of 5G communications, operation within the mmWave spectrum possesses additional challenges and constraints as compared to below 6 GHz communications. Among others, increased atmospheric absorption, greater impact of hydrometeors (such as rain, hail or snow), increased building penetration losses, increased shadowing effect from the presence of human body or the impact of non-ideal surfaces and surface roughness in diffuse scattering components owing to smaller operation wavelength [3]. Regarding the biological and health impacts of the Radio Frequency Electromagnetic Field (RF-EMF) exposure of 5G systems at the mmWave frequency range (6–100 GHz), several studies can be found in the literature [4,5,6,7,8] showing no clear evidence of harmful or potential RF-EMF hazards from the 5G exposure over the general population. Furthermore, it must be remarked that the penetration depth inside the human body will decrease in mmWave frequency bands, especially while increasing the frequency [9,10]. However, RF-EMF research is still being promoted by the World Health Organization (WHO) [11], due to the lack of clear or conclusive evidence to determine whether there are any health consequences from the RF-EMF exposure of 5G systems particularly in mmWaves.

In this work, mmWave wireless channel analysis is presented for a realistic scenario, based on an in-house deterministic Three-Dimensional Ray-Launching (3D-RL) algorithm, in which full topological and material characteristics have been taken under consideration. To this end, specific simulation modules have been implemented in order to consider within the mmWave band, the extraction of frequency domain, time domain, statistical modelling (large scale/small scale) and interference parameters. Estimations for received power levels for several mmWave bands, as a function of antenna beam width and orientation, are obtained for the cases of outdoor as well as indoor coverage. These results in turn can be used to analyze time domain characteristics, such as power delay profiles and delay spread distributions for the complete volume of the scenario. Statistical analysis is also performed, showing the best fitted distribution for different scenarios, as well as an interference analysis, obtaining Signal-to-Noise Ratio (SNR) distributions and error probability for the Single-Carrier Modulation Carrier Scheme 12 (SC MCS-12). The proposed methodology enables the estimation of multiple parameters, such as frequency domain, time domain, small/large scale propagation metrics and interference values, for any location within the full volume of the scenario under test, providing a flexible analysis tool in relation with mmWave-related functionalities and applications.

## 2. Background

Multiple modeling techniques have been proposed, such as the stochastic channel models, empirical models, geometry-based models, and deterministic models [3]. The propagation characteristics of the mmWave band frequencies are different from those of the sub-6 GHz bands. This is due to the high path loss, poor diffraction, high penetration losses and high sensitivity to blocking, among others. Related to the small wavelength, antennas with small dimensions are used in mmWave bands, which promotes the use of Multiple-Input-Multiple-Output (MIMO) systems [12]. Taking these characteristics into consideration, the use of spatially consistent propagation models such as geometric stochastic or deterministic models are preferred for the emulation of the wireless communication channel at these frequency bands. Multiple studies have been performed and channel models have been proposed in order to provide adequate wireless channel characterization, up to the 100 GHz frequency range, such as 3rd Generation Partnership Project–Technical Report (3GPP-TR) 38.901, 5G Channel Model, Mobile and wireless communications Enablers for the Twenty–twenty Information Society (METIS) or Millimeter-Wave Based Mobile Radio Access Network for 5G Integrated Communications (mmMAGIC) [3]. In the case of deterministic techniques, they are mainly based on the use of Ray Launching (RL) or Ray Tracing (RT) (in contrast with full wave electromagnetic approaches) in order to decrease the computational processing of simulations of complex environments. These techniques require accurate and precise information on the shapes, sizes, reflexivity and type of materials that need to be used in the urban environment under study. Formerly, RL and RT were both classified as RT methods, although latterly both methods are differentiated. The dissimilarities are mainly due to different approaches. RL technique principle is based on the shooting and bouncing technique, where thousands of test rays are launched from the transmitter in a solid angle and the true path is determined by searching for the rays arriving at the receiver. By contrast, in classical RT methods the reflected paths by walls and furniture are found by computing the image of the transmitter or of the receiver. Deterministic techniques exhibit a larger computational cost, but at the same time provide precise information in relation to the impact of topological features in wireless channel characterization. This is a particular case of dense transceiver scenarios (such as those provided by D2D and MTC links), in which transceiver distances can be relatively small compared to overall scenario dimensions. Different techniques and algorithms are used to increase the computational efficiency of RT. In the case of [13], the use of a pre-calculated intra-visibility database is proposed, obtaining as a result a reduction in pre-processing time of 90%. 

The advent of 5G systems demands among other requirements the use of new spectral bands, given in Frequency Range 1 (FR1, below 6 GHz) and Frequency Range 2 (FR2, above 6 GHz). Operation within these spectral bands is given as a function of diverse applications, such as vehicular connectivity or ultra-dense network deployments within IoT scenarios [14,15]. Characterization of urban environments for 5G systems, in the mmWave bands, has been proposed with a limited set of RT-based approaches. The lack of information about the characteristics and distributions of the environment is one of the main obstacles facing the implementation of RT techniques in virtual environments. The specific features of buildings in outdoor environments, usually use databases with spatial resolutions in the order of a few meters, as a result, an accurate description of the environment is of imperative significance [16]. Some of the literature has been focused on obtaining statistical descriptions of the channels based on the behavior found through RL modeling [17], where mmWave frequencies such as 28 and 73 GHz were considered in the simulation results. In [18], RT techniques are used together with graph theory concepts. The graph theory is incorporated to establish a propagation graph that considers the environment and its geometry, as well as mobility and distribution of objects. This combination allows us to obtain a semi-deterministic modeling technique where propagation is assessed with accuracy specially in the delay and angular domains.

RT and mmWave have been used to simulate different environments and specific scenarios and applications, such as the human body [19]. The model was implemented on processors using parallel computation to simulate the environment. In [20], the authors combine the measurements of propagation in frequencies from 2 to 28 GHz and extend the study through the use of RT to frequencies up to 73 GHz for urban macro environments. These studies reveal that it is compulsory to have physical measurements where modeling is based on predicting losses, following a measurement-supported channel characterization approach. In [21], the results obtained in a RT simulation are presented, presenting an outdoor scenario at 28 GHz carrier. In this research, a cloud-based RT platform was used, thereby increasing performance. The RT technique has also been used in atypical environments such as high-speed trains [22]. This research is carried out at the 25.25 GHz frequency, and different environments such as urban, rural, and tunnel are used. Results are obtained in parameters such as path loss, Root-Mean-Square Delay Spread (RMS DS) and coherence bandwidth, among others. Another 28 GHz outdoor study, employing RT for analysis, can be found at [23]. In this research, simulations performed in RT software are used, and as a result, a propagation model at 28 GHz is obtained for urban microcellular conditions. Also, at 28 GHz, the work done by [24] presents the results obtained from a simulation based on 3D RT software. The results are evaluated with measurements made at the same location, showing a good approximation with the simulated values. In [25], a RT technique is used to extend the scarce empirically obtained data to analyze the specific characteristics of the communication channel in mmWave bands. The simulation is performed using the shot and rebound technique, where the elements of the environment are simplified, eliminating the effects produced by cars and people. As a result, a propagation model is proposed following a 3GPP spatial channel model methodology based on the data obtained in simulations and measurements. 

Indoor modeling of mmWave propagation has also been carried out with RL techniques. For example, in [26] a 3D-RL technique is shown for a conference room at frequencies above 60 GHz, where a time variant scenario is analyzed. The simulation of the RL technique uses arbitrary polygonal shapes to omit areas that are not important to quantify propagation. Also, analytical techniques are combined with the simulation. Another indoor 3D scenario where simulation is performed using RL techniques is shown in [27], where they have close agreement with measurements for delay spread parameter. In [28,29], the wireless directional propagation channel in an indoor 70 GHz scenario is investigated, with the assistance of RT simulations and measurements. Results such as the angular and temporal dispersion parameters are some of the analyzed results presented, showing good precision. The RT method presents an error of less than 2 dB in the received power, and an RMS delay spread percentage error of about 5%. The work presented in [30], analyzes the behavior of massive MIMO systems at mmWave frequencies in the 26 GHz bands. In this study, a RT simulator is used, which is validated and calibrated, to surpass the limitations on the possible number of array elements. After calibration, the algorithm presents an error of 3.5 dB with respect to the Power Delay Profile (PDP) obtained during the measurement process, showing a good agreement between the measurements and the simulation. In [31], the 60 GHz wireless communications channel is modeled in an indoor environment. In such work, a RL software based on the shot and bounce method is used. Small-scale parameters such as the Power Angular Spectrum (PAS) and the PDP are obtained and analyzed through this technique. Some other indoor RT technique-assisted studies can be found in [32,33,34]. Wireless channel analysis studies have been reported in order to consider other aspects, such as vehicular channel characterization and impact of infrastructure and communication link types [35,36,37], the impact of topological impact in wireless channel characterization [38,39], the effect of rain in mmWave 5G links [40], the impact of EMF constraints in 5G wireless system design and deployment [41] or the use of artificial intelligence assisted techniques in order to enhance functionalities such as beamforming or spectral handling, among others [42,43]. A summary of wireless channel characterization studies is presented in Table 1.

## 3. Wireless Channel Characterization

### 3.1. Deterministic 3D Ray Launching Algorithm

In order to perform wireless channel analysis, an in-house implemented 3D-RL algorithm has been used for the spatial radio propagation characterization of different scenarios (outdoor and indoor, as well as the transitions among them) at the frequency bands proposed for the new generation of wireless communication systems 5G, specifically 28 GHz, 30 GHz and 60 GHz bands. The in-house 3D-RL algorithm, based on Geometrical Optics (GO) and the Uniform Theory of Diffraction (UTD), has been widely validated in the literature for frequencies below 6 GHz, for urban complex environments, smart health systems, context aware environments, ITS and interference analysis [44], among other applications. 

**Figure 1:**
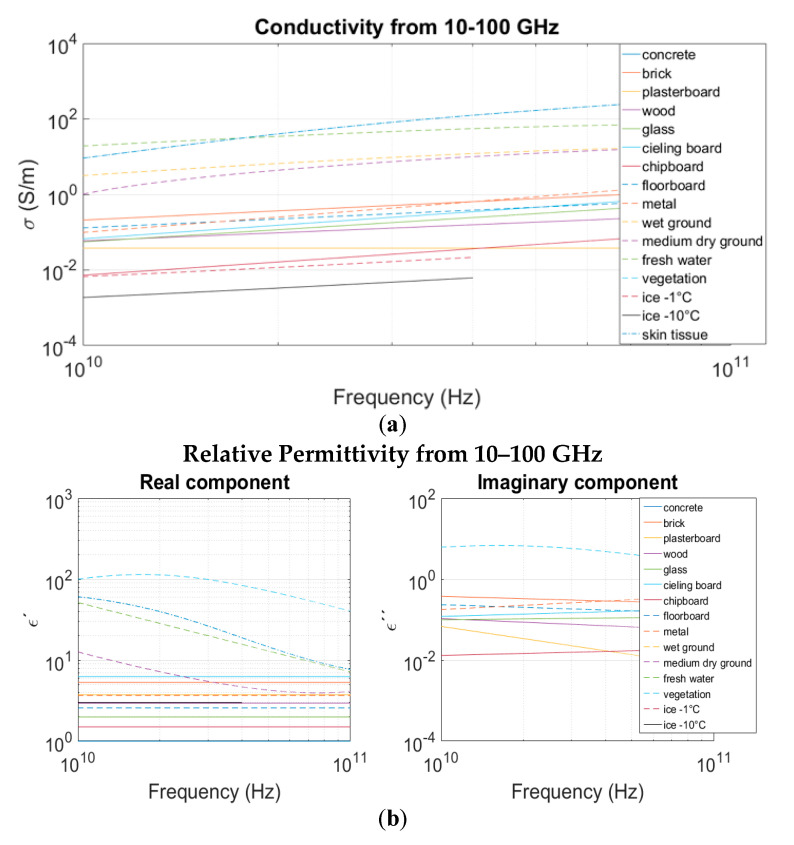
(**a**) Conductivity as a function of frequency for different materials, (**b**) Relative permittivity as a function of frequency for different materials.

In this work, a new module has been developed to be implemented in the in-house RL algorithm to analyze radio wave propagation for mmWave frequencies. RL techniques provide a numerical approximation for the electromagnetic waves behavior as they interact with the environment. Electromagnetic wave propagation mechanisms such as reflection, refraction and diffraction can be estimated from the geometry of the objects and dispersive material properties. The International Telecommunications Union (ITU) provides models for the electric conductivity and permittivity, as functions of frequency, for different building materials for the 1–100 GHz frequency band in Recommendation Please include: International Telecommunication Union-Radiocommunication (ITU-R) P2040-1 [3]. These models were obtained from experimental measurements. The materials included in ITU-R P.2040-1 are the following: vacuum/air, concrete, brick, plasterboard, wood, glass, ceiling board, chipboard, floorboard and metal. Recommendation ITU-R P.527-3 [17] provides plots for conductivity and permittivity of ground, water and ice for the 10 kHz–100 GHz frequency band. Regression models were obtained from the plotted data in ITU-R P.527-3 for the 10–100 GHz frequency band. Models of the electrical properties of vegetation were obtained from [18]. All the models mentioned above were added to the mmWave module in the in-house 3D-RL simulator, as well as the atmosphere absorption phenomena, which is more relevant at these higher frequencies than at the below 6 GHz frequency spectrum allocation [45]. Figure 1 shows the electric conductivity and permittivity for different building materials, ground and vegetation as a function of frequency, in the frequency range of 10–100 GHz, for real as well as imaginary components. 

**Figure 2:**
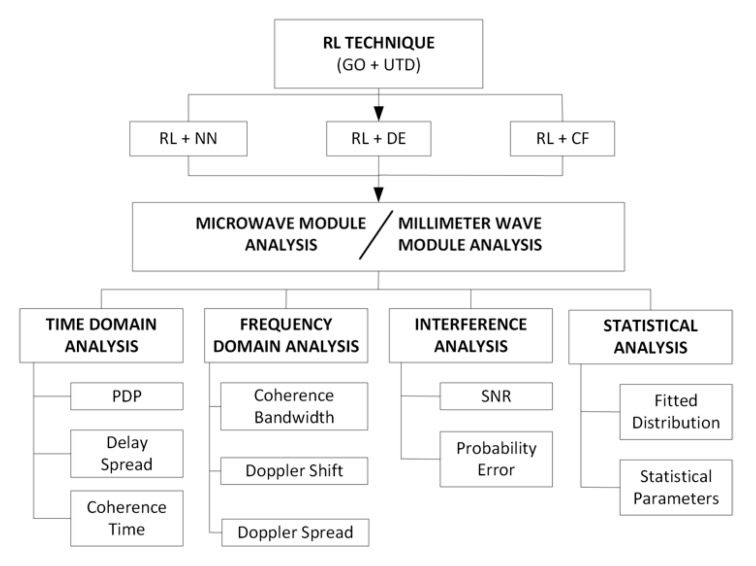
Schematic view of the Three-Dimensional Ray-Launching (3D-RL) algorithm implemented modules.

The option of considering different beamforming capabilities is also included, leading to the possibility of analyzing different types of antennas with adaptive radiation patterns, employed for connectivity as well as for initial access purposes. Figure 2 shows a schematic view of the different modules of the current RL tool, which have been implemented ad-hoc to provide full interoperable capabilities within the code. The 3D-RL estimation engine can employ a hybrid simulation with a Neural Network (NN) ray interpolator module [46], 2D Diffusion Equation (DE) approach [47] or Collaborative Filtering (CF) database learning technique [48] to decrease the computational cost, with accurate results, depending on the dimensions of the considered scenario. Moreover, depending on the frequency under analysis, the microwave or mmWave analysis module will be used in the simulation. Figure 2 also shows different results which can be obtained from the simulation, in terms of time and frequency domain analysis, and statistical and interference analysis.

### 3.2. Scenario Description

**Figure 3:**
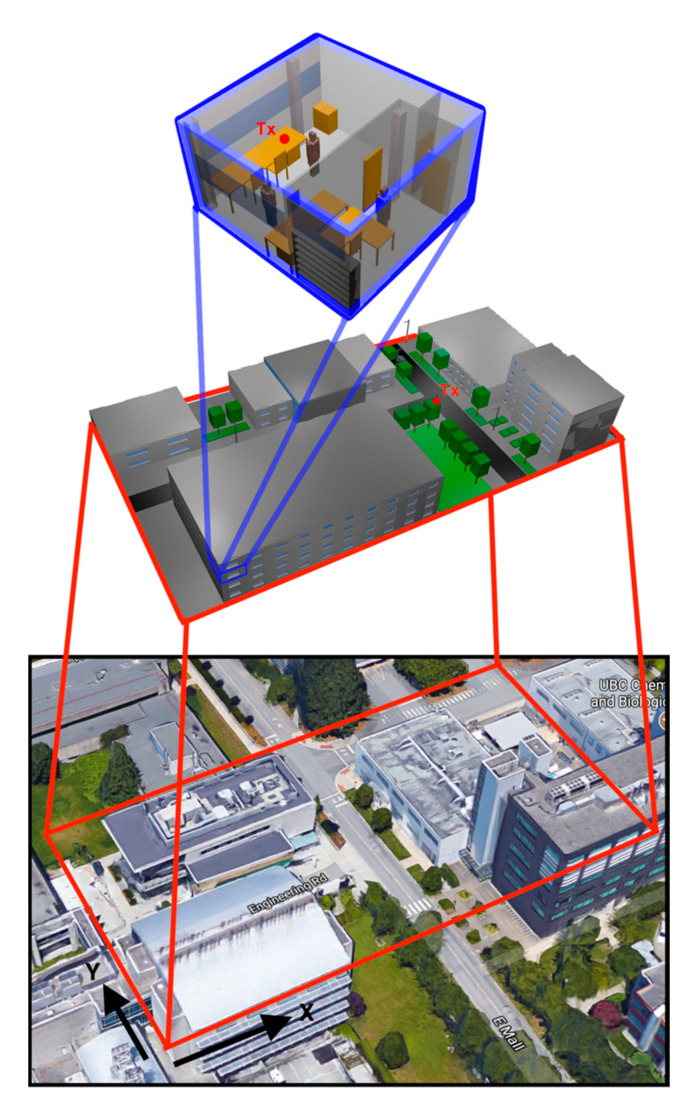
Real aerial view of the considered scenario alongside the schematic representation of the simulated scenarios (outdoor and indoor) and the employed 3D ray-launching parameters for each of the considered scenarios.

In order to perform the channel characterization of mmWave communications within 5G frequency bands, two different scenarios have been considered, a complex outdoor scenario and an indoor scenario, which are shown in Figure 3. The outdoor scenario corresponds to a real location within the campus of the University of British Columbia (UBC), Canada. The scenario can be considered a complex scenario, with different zones, two main roads, different types of buildings with different heights and inhomogeneous vegetation environment, in which the complete volumetric description has been considered, thanks to the inherent volumetric nature of the 3D-RL code. The proposed scenario can represent potential locations for the implementation of context-aware services enabled by 5G systems, such as ITS or D2D communications.

The aerial view of the scenario can be seen in Figure 3 (bottom), the schematic representation (middle) and the indoor considered scenario (top). The dimensions of the outdoor scenario are 135 m by 70 m by 18 m, and the dimensions for the indoor scenario were 5.84 m by 6.24 m by 3.5 m. The complete topology as morphology of the scenarios was taken into account, in terms of dimensions and locations. All the dispersive material properties (i.e., conductivity and dielectric permittivity) as a function of the corresponding frequency of operation were also considered, as explained in the previous section. Frequency bands of 28 GHz and 60 GHz were selected as potential operating bands for future 5G deployments, whereas the 30 GHz band was also analyzed for radio propagation validation purposes with experimental measurement results. Holistic system operation is given by considering indoor operation, which can be provided by 802.11ad hot-spots located within indoor environments. As detailed in Figure 3, such systems were also considered, within an office in which full constructive details, as well as the inclusion of human body models were included. Simulation parameters for each one of the analyzed scenarios are shown in Table 2. 

**Figure 4:**
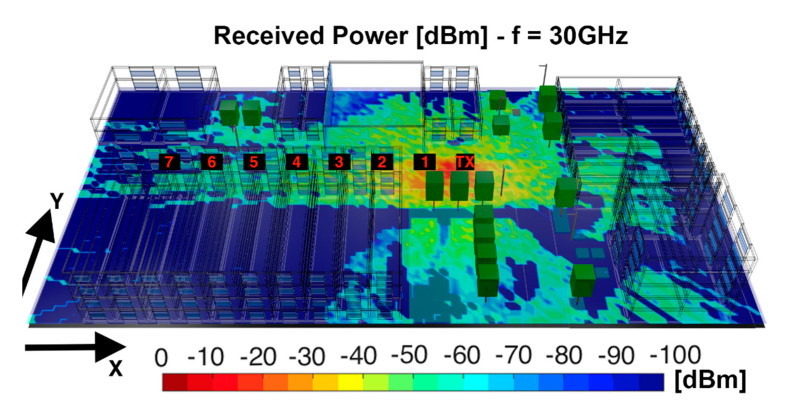
Bi-dimensional plane of received power [dBm] for a 1.3 m height with the position of the measurements points and transmitter position highlighted.

In the case of outdoor analysis, unitary volume analysis mesh resolution was fixed to 1 m, whereas in the case of the indoor scenario, it was fixed to 0.1 m. The total number of allowed reflections until extinction was fixed to 6 reflections. Simulation parameters were selected following algorithm convergence analysis, to provide an adequate trade-off between accuracy and required simulation time [49]. For the outdoor validation scenario (Case I), the transmitter position was placed at (X = 78.7 m, Y = 40.3 m, Z = 1.3 m), emulating a User Equipment (UE) placed in an outdoor location. In addition, for the outdoor simulation scenario (Case II), the transmitter position was placed at (X = 87.8 m, Y = 32.9 m, Z = 4 m), in a streetlight, to analyze the link between the infrastructure and a device that could be carried by people, or Vehicle-to-Infrastructure (V2I) communications. For the indoor scenario (Case III), the transmitter position was at (X = 3.5 m, 5.8 m, 1.05 m), emulating a device placed above the table for WLAN 802.11ad coverage. The different location of the transmitter antenna for the outdoor and indoor simulation scenario (Cases II and III) are shown in Figure 3 as a small red circle. The position of the transmitter for the outdoor validation scenario (Case I) is shown in Figure 4, indicated by TX. Wireless channel analysis was performed considering large-scale as well as small-scale propagation conditions, which will be described in the following sections.

### 3.3. Large-Scale Propagation

The large-scale wireless channel characteristics were evaluated for 28 and 30 GHz frequencies for the outdoor scenario, and 60 GHz frequency for the indoor scenario. First, the outdoor validation scenario was analyzed and compared with real measurements. Figure 4 shows for 30 GHz the bi-dimensional plane of the Received Signal Strength Indicator (RSSI) for the receiver located at 1.3 m Above Ground Level (AGL), obtained with the 3D-RL code, following the parameters shown in Table 2 for Case I. From the RSSI figure, it can be seen that the morphology and topology of the scenario have a great impact in radio propagation. A high signal absorption of the buildings and clutter can be observed in the scenario. 

**Figure 5:**
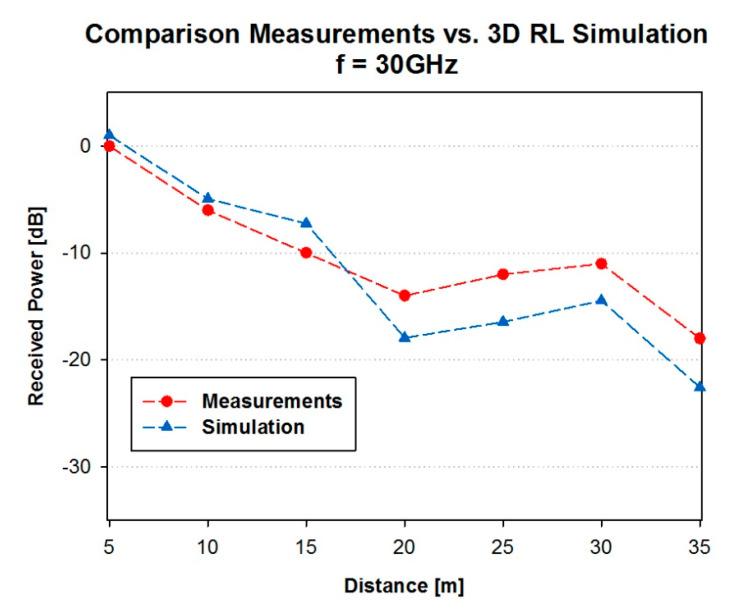
Comparison measurements vs. 3D-RL simulation.

In order to validate the simulation results, a campaign of measurements conducted in the same scenario, which corresponded with a street canyon location at the campus of University of British Columbia (UBC), was used [50]. The measurement platform used was a Vector Network Analyzer (VNA) based channel sounder. A 30 GHz channel sounder with 1 GHz bandwidth, 1 ns temporal resolution and high dynamic range was used for that purpose. The VNA was operating at 1.2 GHz center frequency with a 1.2 GHz/30 GHz up and down converters. An omnidirectional bi-conical antenna with 3 dBi gain was used at the transmitter side, which transmitted a 36 dBm radio frequency signal. As for the receiver, a highly directional, vertically polarized horn antenna with 26 dBi gain and 10° half power beamwidth, was used. The position of the transmitter and receiver were 1.3 m AGL. Measurements were performed by moving the receiver in 5 m steps apart from the transmitter, which are depicted in Figure 4. Figure 5 shows the comparison between the experimental measurements and simulation results for the considered measurement points. Good agreement is observed among the results, with a mean error of 3.04 dB and standard deviation of 1.5 dB, validating the proposed simulation methodology for the case of a complex scenario, with outdoor as well as indoor operating conditions.

**Figure 6:**
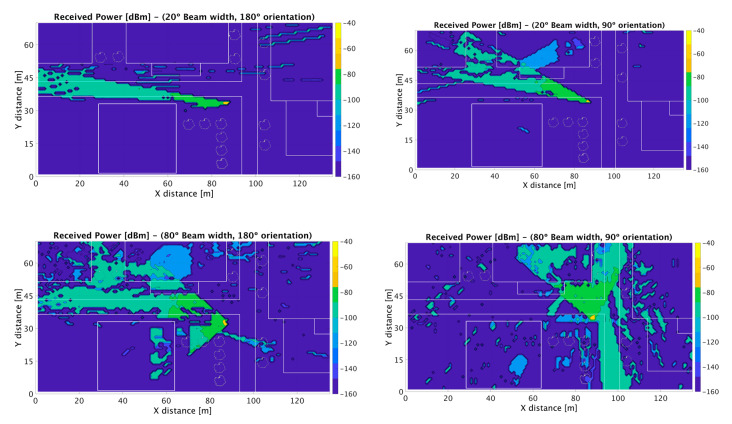
Simulated mmWave power distribution for outdoor coverage (at 28 GHz, beam width values of 80° and 20°, with 180° and 90° orientation), under simulation parameters defined in Table 2.

**Figure 7:**
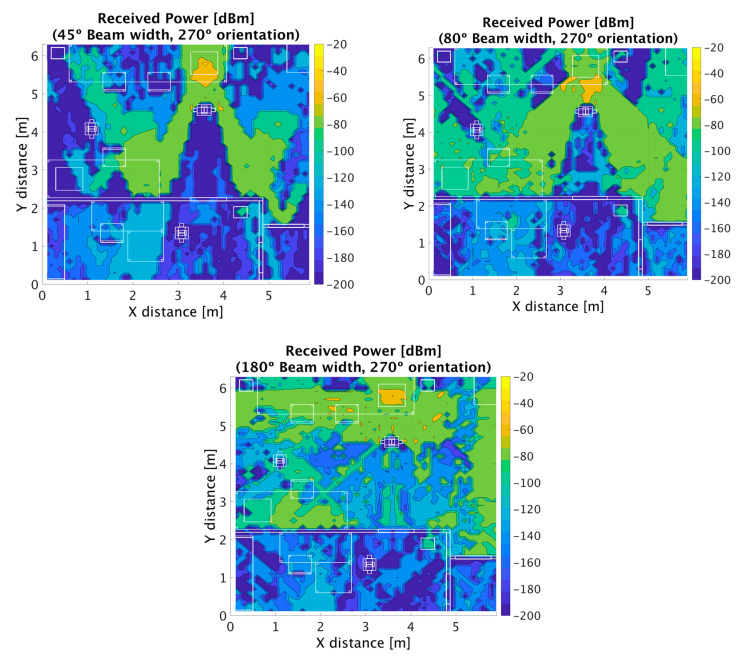
Simulated mmWave power distribution for indoor coverage (at 60 GHz, beam width values of 45°, 80° and 180°, 270° antenna orientation), under simulation parameters defined in Table 2.

Once the simulation method was validated in the UBC scenario at 30 GHz frequency band, analysis of 5G operation at 28 GHz and 60 GHz was performed, employing the simulation parameters in Table 2 for Cases II and III. The scenario was implemented with full building details (indoor rooms, furnishings and separations) as well as outdoor detail. In this way, urban microcells (UMi) as well as indoor WLAN-based femtocells can be studied. The proposed scenario can also be employed to analyze part of the proposed ITS use cases, mainly inter-car, intra-car and infrastructure to infrastructure communications [51]. Different beamwidth values were considered, owing to potentially different coverage areas, as well as to different initial access mechanisms (such as iterative search, in which initial phases require transmission over wider beamwidth sectors) [52]. The outdoor transceiver was located on the right-hand side of the scenario, with 80° antenna beamwidth (initially oriented at 180° and then steered to 90°) and a 20° beamwidth (initially oriented at 180° and then steered to 160°). The proposed coverage could adequately provide ITS based communication capabilities within the urban canyon in which the street was located. In the case of the indoor scenario, WLAN 802.11 ad coverage was considered, mainly for wider beamwidths although potential beam orientation was also considered (beamwidths of 45°, 80° and omni-directional radiation pattern). Bi-dimensional distributions of received power levels for cut planes at a height of 1.3 m are shown in Figure 6 for the outdoor scenario and in Figure 7 for the indoor scenario, in which beamwidth variation clearly modifies overall coverage levels. 

In the case of outdoor 180° orientation, an urban canyon-type behavior can be observed, owing to the distribution of the buildings along the central street, with higher levels along the street in the case of 20° beam width radiation pattern. In the case of indoor operation, blocking effects are clearly visible, due to the presence of structural elements such as walls. Human body models have also been implemented ad hoc for inclusion within the 3D-RL code, in which a relevant shadowing effect is also visible.

### 3.4. Small-Scale Propagation

**Figure 8:**
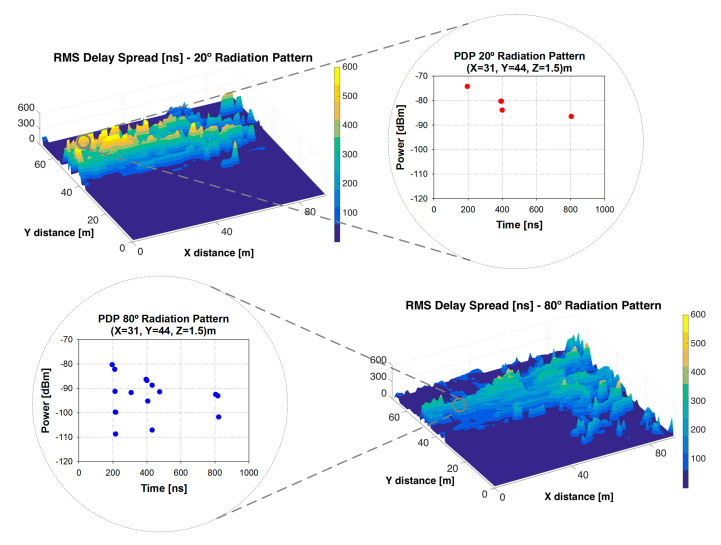
Root-mean-square (RMS) delay spread for 20° and 80° radiation pattern with 180° orientation, for a height of 1.3 m with its associated Power Delay Profile for a specific point in the scenario.

Millimeter-wave frequency signals also observe a large percentage of multipath contributions since objects traditionally acting as scatterers, now become reflectors at mmWave frequencies, and may induce significant multipath effects. Small-scale effects have recently been analyzed in mmWave bands, analyzing effects of diffracting corners and proposing antenna models based on double knife edge diffraction models [53]. To gain an insight into the impact of multipath propagation in the considered scenario, different small-scale propagation phenomena were extracted from simulations results and are presented in Figure 8, which shows the PDP and RMS DS for 28 GHz for the considered outdoor simulation scenario (Case II) for different radiation patterns of the transmitter antenna (20° and 80° angular beamwidth in the radiation pattern of the principal lobe, with 180° orientation for both). The PDP represents the different multipath components which arrive at different times with its associated received power. Figure 8 (top) represents the RMS DS in nanoseconds for a bi-dimensional plane at 1.3 m AGL, with a PDP depicted for a considered specific point of the scenario. From the RMS DS, certain areas exhibit sparse ray density, given by the use of transmitters with directive radiation patterns, in accordance with general RL behavior related with inherent limitations linked with angular resolution versus distance. It is also observed that the maximum time span is approximately 800 ns, in line with previous simulation as well as measurement results derived in multiple indoor as well as outdoor scenarios [51]. 

Figure 8 (bottom) represents the PDP and the RMS DS changing the radiation pattern of the transmitter antenna to 80°. It is observed that in this case, multipath propagation increases, as it is shown in the PDP figure. The noise threshold used to depict these results has been −120 dBm. It can be seen that the values of the RMS DS are approximately in the order of hundreds of nanoseconds in the majority of the spatial samples, which is coherent compared with real measurements for outdoor scenarios. As previously stated, these results depend heavily on the morphology and topology of the considered scenario, the antenna pattern model, frequency of operation of the system under analysis, as well as other phenomena, such as weather or atmospheric effects. All these effects must be taken into account before the design and implementation of mmWave wireless networks, depending of the considered scenario and the system application. Time delay estimations related to mmWave propagation are relevant in order to derive overall time delay and RTT, taking into account stringent 1 ms required values to achieve immersive Internet properties [1]. The results presented in Figure 8 provide full spatial characterization of PDP as well as delay spread values, which in turn provides information that can be employed to analyze further system parameters, such as restrictions in the application of analog beamforming techniques [54].

**Figure 9:**
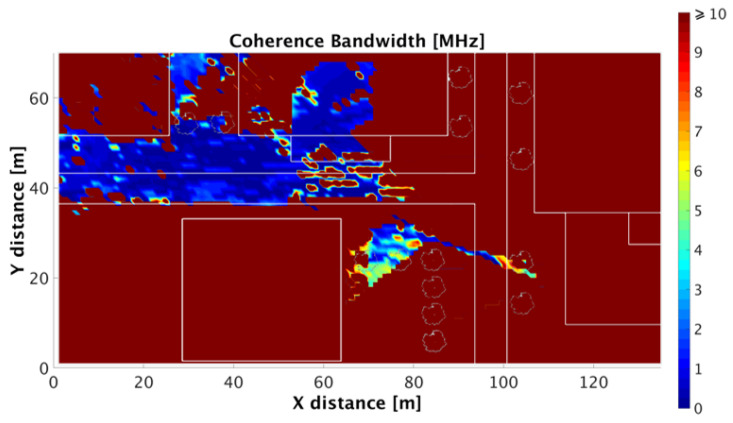
Coherence bandwidth for the outdoor scenario (80° beam width and 180° antenna orientation) at the bi-dimensional plane of 1.3 m height.

To gain insight in the frequency domain channel description, Coherence Bandwidth (CB) has been characterized from the RMS DS for the considered outdoor scenario (Case II). The RMS DS and CB depend on the exact multipath structure. The CB has been defined as the bandwidth over which the frequency correlation function is above 0.9, then the CB is approximately $B_{c}\approx 1/50\sigma_{\tau }$, see [55], where $\sigma_{\tau }$ is defined as the RMS DS. Figure 9 shows the CB for the specific case of the outdoor simulation scenario (Case II), with 80° beam width and 180° orientation, showing values which vary from 0.01 to 10 MHz, which is coherent as shown in [56]. It can be seen that there is a great variability in the scenario, and the higher values of CB are in the sections where the received power is lower than the noise threshold considered. The range of frequencies which includes components with strong potential for amplitude correlation is higher in the areas with small values of RMS DS and when the distance from the transmitter is larger. Therefore, a receiver in those areas, with a high CB, (e.g., CB upper threshold >10 MHz), potentially can experience stronger multipath interference.

### 3.5. Statistical Analysis

Statistical analysis of the received power components caused by multipath trajectories has been carried out for both outdoor and indoor scenarios (Cases II and III), at 28 and 60 GHz frequencies. The methodology followed has consisted in fitting five distributions to the simulated data. The first distribution considered was the Nakagami distribution, whose probability density function is given by [57],

(1) $$f\left(\;x\;|\;\mu ,\;\omega \right)=\frac{2\mu^{\mu }}{\Gamma \left(\mu \right)\omega^{\mu }}x^{2\mu -1}e^{-\frac{\mu }{\omega }x^{2}},\;\forall \;x\;\geq 0,$$

where *µ* and *ω* are the shape and scale parameters respectively.

The second fitted distribution was the Rayleigh distribution, with probability density function given by [55],

(2) $$f\left(x\;|\;\beta \right)=\frac{1}{\beta^{2}}xe^{-\frac{x^{2}}{2\beta^{2}}},\;\forall \;x\;\geq 0,$$

where *β* is the parameter of the Rayleigh distribution.

The third distribution considered for the analysis was the Weibull distribution, whose probability distribution is given by [58],

(3) $$f\left(x\;|\;a,b\right)=\frac{b}{a}{\left(\frac{x}{a}\right)}^{b-1}e^{-{\left(\frac{x}{a}\right)}^{b}},\;\forall \;x\;\geq 0,$$

where a and b are the scale and shape parameters respectively.

The fourth distribution fitted to the scenarios was the Log-normal distribution, whose probability distribution is given by [55],

(4) $$f\left(x\;|\;\mu ,\sigma \right)=\frac{1}{x\;\sigma \sqrt{2\;\pi }}e^{-{\frac{\left(lnx-\mu \right)}{2{\;\sigma }^{2}}}^{2}},\;\forall \;x\in \mathbb{R},$$

where *μ* is the mean of lnx and *σ* is the variance parameter.

The fifth distribution fitted to the scenarios was the Gamma distribution, whose probability distribution is given by [55],

(5) $$f\left(x\;|\;a,b\right)=\frac{1}{\Gamma \left(a\right)b^{a}}{\left(x\right)}^{a-1}e^{-\frac{x}{b}},\;\forall \;x\;\geq 0,$$

where a and b are the scale and shape parameters respectively.

**Figure 10:**
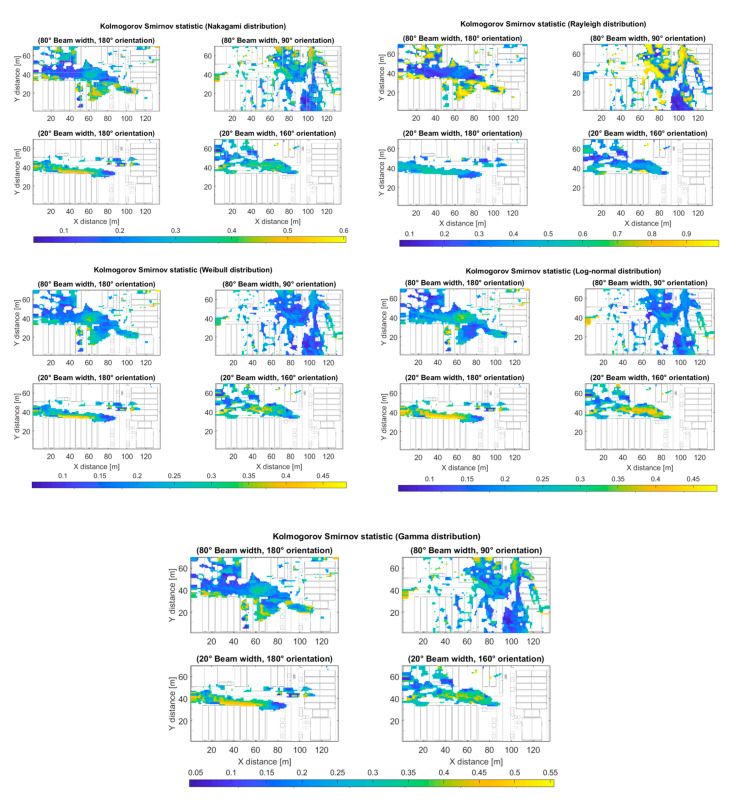
Kolmogorov–Smirnov statistic between the data from simulation and Nakagami distribution (**top left**), Rayleigh distribution (**top right**), Weibull distribution (**center left**), Log-normal distribution (**center right**), and Gamma distribution (**bottom**) for the outdoor scenario and different transmitter antenna configurations.

It is worth mentioning that for all the distributions considered, the random variable x is the received power due to multipath, measured in mW. These five distributions have been considered for being the most commonly used for the analysis of radio wave propagation in complex environments [55,58]. The maximum likelihood estimators for all the distributions parameters were found for each cuboid that contained at least thirty received rays, for both scenarios. We also tried to fit the Rician distribution, but we were not able to find convergence of the non-centrality parameter of the distribution by the maximum likelihood method because of the skewness of the simulated data. To decide which distribution best fitted the data, a comparison between the goodness of fit measure for each distribution was performed. The goodness of fit measure used was the Kolmogorov–Smirnov statistic between the simulated data and the fitted distributions. The Kolmogorov–Smirnov statistic is a metric of the distance between a given reference cumulative distribution and the empirical cumulative distribution obtained from the simulated data. The Kolmogorov–Smirnov statistic is given by,

(6) $$D_{n}=\underset{x}{\sup}\left|F_{n}\left(x\right)-F\left(x\right)\right|,$$

where $D_{n}\in \left[0,1\right],\;F_{n}\left(x\right)$ is a reference cumulative density function, either Nakagami, Rayleigh, Weibull, Log-normal or Gamma distribution; and $F\left(x\right)$ is the empirical cumulative density function of the simulated data. Note that the smaller the value of the Kolmogorov–Smirnov statistic, the better the goodness of fit of the proposed distribution and the data. Figure 10 (top left) shows the Kolmogorov–Smirnov statistic between the fitted Nakagami distribution and the data for the outdoor simulation scenario (Case II) for different receiver antenna configurations at 28 GHz frequency. The maximum values of the statistic are around 0.6 and correspond to the regions where fewer rays were received and, therefore, there is more statistical uncertainty. Figure 10 (top right) shows the statistic between the fitted Rayleigh distribution and the data for the same scenario (Case II). Note the higher scale of values when compared to the Nakagami case. Figure 10 (center left) shows the statistic between the fitted Weibull distribution and the data for the same scenario (Case II). Note the low scale of values of the Kolmogorov–Smirnov statistic for the 20° beam width cases. Figure 10 (center right) shows the statistic between the fitted Log-normal distribution and the data for the same scenario (Case II). Note the low scale of values of the Kolmogorov–Smirnov statistic for the 80° beam width cases. Figure 10 (bottom) shows the statistic between the fitted Gamma distribution and the data for the same scenario (Case II). Note the values of the Kolmogorov–Smirnov statistic are comparable to the Nakagami distribution case. 

**Figure 11:**
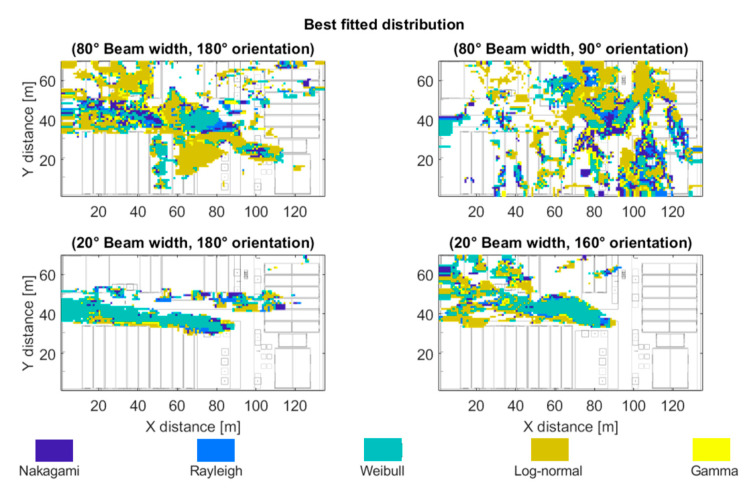
Best fitted distribution for the outdoor scenario (at 28 GHz, beam width values of 80° and 20°, with 180° and 90° orientation) at the bi-dimensional plane of 1.3 m height.

Taking into account these results of the Kolmogorov–Smirnov statistic, an analysis has been done to know the best fitted distribution of each spatial sample of the scenario, related with the transmitter position at the bi-dimensional plane of 1.3 m height, emulating different UE in the outdoor scenario. Figure 11 highlights in navy blue the regions where the best fitted distribution was the Nakagami distribution. The light-blue color represents regions best fitted by the Rayleigh distribution. Areas in which the best fitted model was the Weibull distribution are highlighted in green. Dark and light-yellow highlight the Log-normal and Gamma distributions respectively. It can be seen that the Weibull distribution is dominant for the narrow 20° beam width receiver antenna configurations, while the Log-normal distribution fits best the wider 80° beam width receiver antenna configurations for this specific scenario at the frequency under analysis.

**Figure 12:**
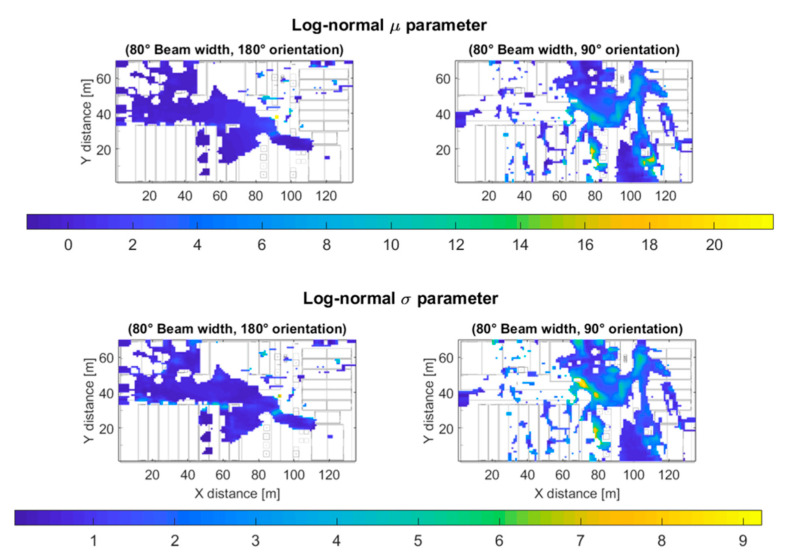
Log-normal distribution’s *µ* (**top**) and σ (**bottom**) parameters for the 80° beam width outdoor scenarios.

Figure 12 displays the values of the μ parameter (top) of the fitted Log-normal distribution for the outdoor scenario and 80° beam width (Case II). The parameter μ of a Log-normal distribution can be used as a large-scale path-loss and fading figure to describe the power loss degree (or level) experienced by a signal which propagates in a multipath channel [59]. The lower the value of the μ parameter, the greater the power loss, note how the value of *μ* decreases with the distance to the transmitter antenna. The σ parameter (bottom) describes the dispersion of the received power components, the greater the σ value, the greater the received power ray’s dispersion. Note the greater dispersion for the 90° antenna orientation case, that was due to the presence of a nearby building and the scattered and refracted rays caused by it, which cause Non-Line-of-Sight (NLoS) communication.

**Figure 13:**
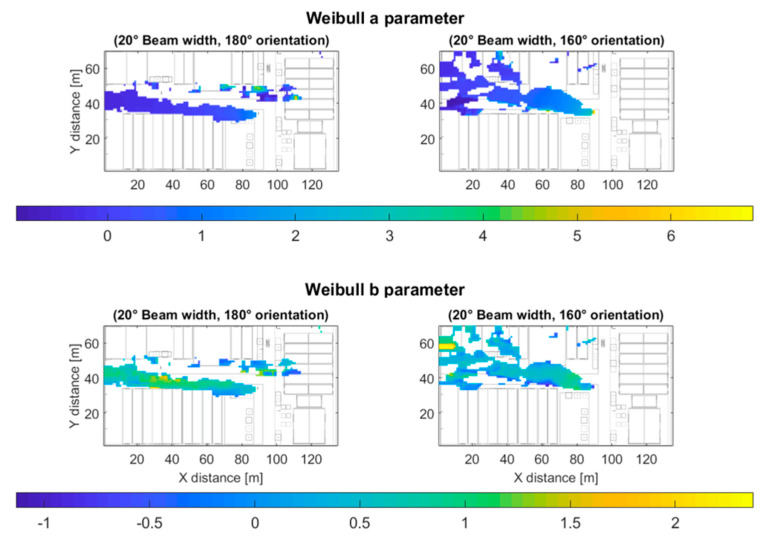
Weibull distribution’s a (**top**) and b (**bottom**) parameters for the 20° beam width outdoor scenarios.

Figure 13 shows the values of the a parameter (top) of the Weibull distribution, for the specific case of 20° beam width. The a parameter controls the scale of the Weibull distribution, and can be associated with the large-scale path-loss and fading power losses. Note that the farther the distance to the transmitter antenna, the lower the value of the a parameter. The b parameter (bottom) of the Weibull distribution controls the shape of the distribution, for lower values of the b parameter (e.g., $b\in \left[1,2\right]$, as in the results shown in Figure 13), the distribution of the received power is skewed to the lower frequencies side, as the value of b increases, the distribution becomes less asymmetrical.

**Figure 14:**
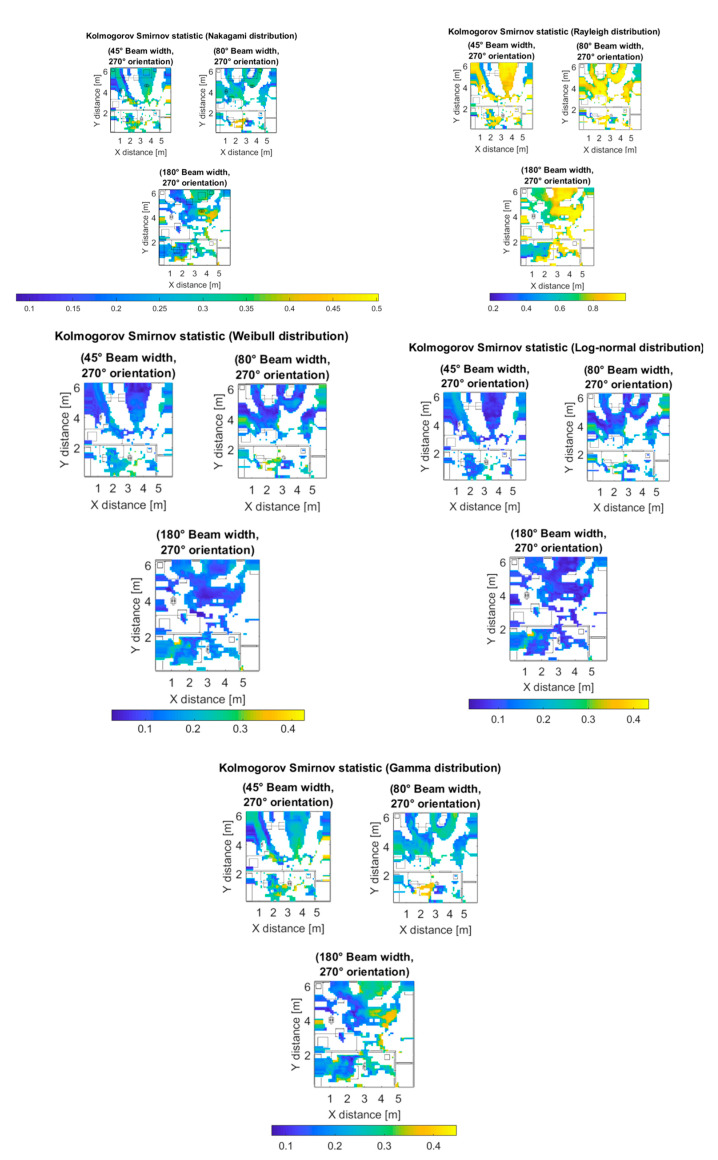
Kolmogorov–Smirnov statistic between the data from simulation and Nakagami distribution (**top left**), Rayleigh distribution (**top right**), Weibull distribution (**center left**), Log-normal distribution (**center right**), and Gamma distribution (**bottom**), for the indoor scenarios.

**Figure 15:**
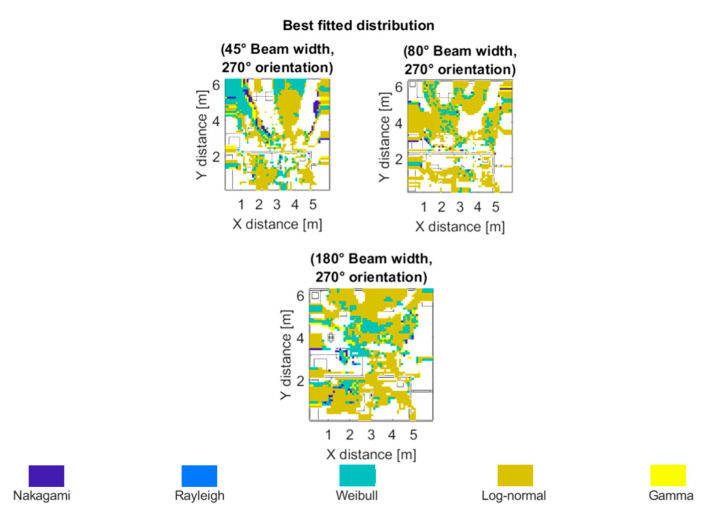
Best fitted distribution for the indoor scenario.

After analyzing the outdoor scenario, the same statistical analysis was performed for the indoor scenario at 60 GHz frequency. The values of the Kolmogorov–Smirnov statistic between the simulated data and the fitted Rayleigh, Nakagami, Weibull, Log-normal, and Gamma distributions can be seen in Figure 14, for the indoor scenario (Case III) for the different beam widths considered. As can be seen from Figure 15, the best fitted distribution was the Log-normal distribution for all the analyzed cases.

**Figure 16:**
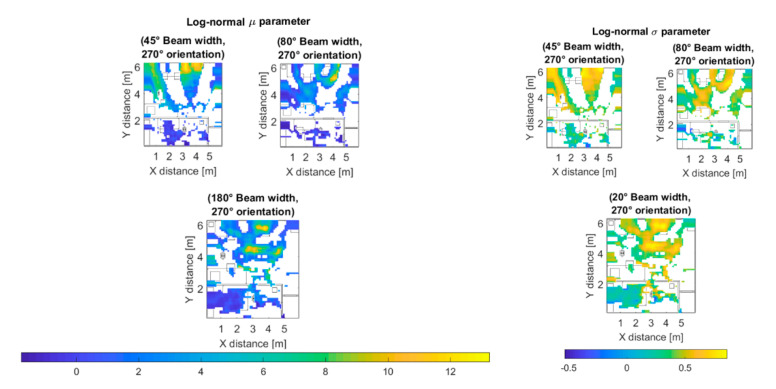
Log-normal distribution’s *µ* (**left**) and *σ* (**right**) parameters for the indoor scenario.

Figure 16 shows the *μ* and *σ* parameters of the fitted Log-normal distribution for the indoor scenario at 60 GHz frequency. It can be observed the high level of fading in this specific scenario at the considered operation frequency.

### 3.6. Interference Analysis

**Figure 17:**
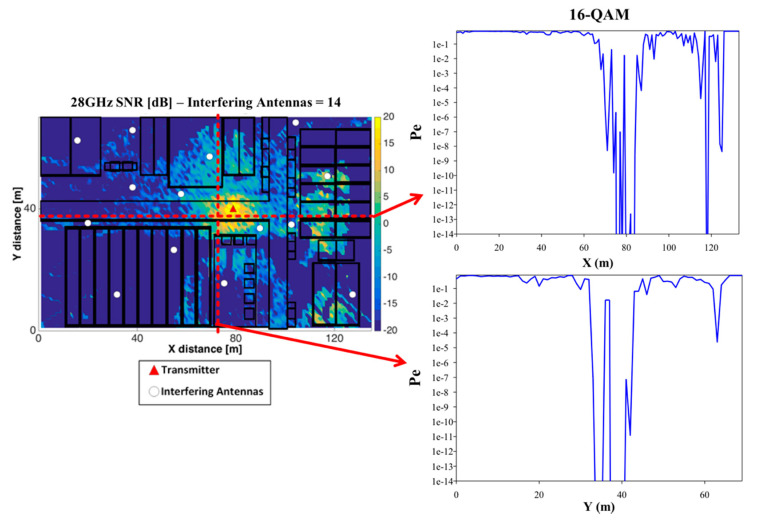
Signal-to-noise ratio [dB] for a bi-dimensional plane of the considered outdoor scenario with 14 interfering antennas (**white dots**) and its associated Bit Error Probability (BER) for two transmitter-receiver (TX-RX) linear radials (**red dashed lines**) along *X*-axis and *Y*-axis for a Single-Carrier Modulation Carrier Scheme 12 (SC MCS-12), 16-QAM modulation scheme.

Once wireless channel characterization can be estimated at the proposed mmWave frequency bands, interference impact has been analyzed by placing a set of potential transceivers within the outdoor simulation scenario (Case II), considering worst case conditions (i.e., uncoordinated co-channel operation). Simulation parameters are given in Table 2, in which Single-Carrier Modulation Carrier Scheme 12 (SC MCS-12) is employed, corresponding to a highly restrictive case in terms of receiver sensitivity requirements. An operating transmitter has been located within the scenario, as well as a set of 14 interfering sources, in different random locations, indicated schematically in Figure 17. Bi-dimensional SNR ratio distribution for a cut plane of height h = 1.3 m is depicted, in which results have been computed for the complete scenario. Considering the highest order modulation employed in MCS-12 (i.e., SC 16-Quadrature Amplitude Modulation (QAM) modulation scheme, highly restrictive), values of probability of error, considering maximum likelihood receiver have been estimated for the complete scenario volume. As an example, the variation for two different linear transmitter-receiver (TX-RX) radials have been depicted in Figure 17 with its associated Bit Error Probability (BER). It can be seen that quality degradation is strongly dependent on a transceiver to device distance, with large variations over small distances, consistent with previous experimental observations [3]. Moreover, despite the fact that shorter transmission links in principle provide higher SNR values and hence, lower error probabilities, non-uniform interference distribution as well as strong fading and shadowing effects can modify coverage/capacity relations. Lower MCS values will enhance QoS metrics, as well as the use of Orthogonal Frequency-Division Multiplexing (OFDM)-based access schemes.

## 4. Conclusions

Future 5G deployments will enable multiple services, including high transceiver densities inherent to D2D and MTC connections, within an IoT framework. The large amount of potential simultaneous transceivers under operation and increased bit rates require the analysis of QoS metrics related with coverage/capacity requirements, interference levels and delay values. In this work, the channel characterization in terms of large-scale propagation, small-scale propagation and interference analysis of mmWave wireless networks for different frequencies has been presented in an outdoor complex scenario, with outdoor and indoor operation, based on a deterministic multi-module 3D-RL code. The impact of beam width, frequency band allocation, MCS election, antenna orientation and node location modify signal as well as interference distributions within the full volume of the scenario under analysis. Effects such as signal blockage given by walls or shadowing effects by the human body are relevant, particularly in the case of indoor operation. Topological dependence of QoS can be adequately assessed employing the proposed deterministic simulation approach, providing optimized network configuration in terms of node layout and antenna configuration, providing a flexible methodology that can be adapted to a wide range of scenarios under analysis. The proposed analysis can be extended to large-density deployments, such as device-to-device (D2D) and machine-type communication (MTC) links, with high accuracy levels.

## Figures and Tables

**Table 1:** Overview of wireless channel characterization studies.

Ref	Description	Results	Freq.
[17]	3D mmWave statistical channel model	Extraction of a statistical channel impulse response model to obtain spatio-temporal characterization	28 GHz/73 GHz
[18]	Semi-deterministic modeling based on ray tracing (RT) and graph theory	Multipath radio propagation is modelled with the aid of a graph theory-based approach, in which scatterers are modelled with realistic scenario elements.	3.8 GHz/60 GHz
[22]	Wireless channel characterization in Fifth-Generation (5G) mmWave railway communications	Wireless channel characterization for different types of scenarios of operation (urban, rural, tunnel) are performed with the aid of RT and measurement results.	25.25 GHz
[23,24]	Micro-cell urban (UMi) wireless channel modeling	Path loss model parameters are proposed employing random variables to consider channel dynamics. 3D RT is employed in [16] to obtain UMi wireless channel characterization	28 GHz
[28,29,30,31,32,33,34]	Indoor characterization	Deterministic RT techniques are employed to characterize directional beam forming behavior in small indoor environments. Multiple-input-multiple-output (MIMO) behavior is analyzed, as well as multiple models proposed for different frequency ranges.	5 GHz/28 GHz/31 GHz/70 GHz/90 GHz
[35,36,37]	Vehicular wireless communication channel characterization	Multiple aspects such as V2V/V2I wireless channel characterization or the impact of urban infrastructure and tunnels is addressed	mmWave/79 GHz/300 GHz
[38,39,40,41]	Topological and environmental impact in wireless channel characterization	Topological aspects, such as scenario type and beamforming characteristics are analyzed, the impact of environmental factors such as rain and the consideration of EMF compliance in wireless planning and analysis are described.	FR1/32 GHz/73 GHz/83 GHz
[42,43]	Application of artificial intelligence (AI) techniques in 5G mmWave wireless channel characterization	Different AI-based techniques are described in order to enhance functionalities such as beam forming or interference analysis, related with wireless channel characterization	Generalizable

**Table 2:** Three-dimensional ray-launching simulation parameters.

Parameters	Outdoor Validation (Case I)	Outdoor Simulation (Case II)	Indoor Simulation (Case III)
Transmitted Power	36 dBm	10 dBm	10 dBm
Operation Frequency	30 GHz	28 GHz	60 GHz
Antenna beam width	Omnidirectional Monopole	80°/20°	180°/80°/45°
Transmitted data rate	100 Mbps	4.62 Gbps	4.62 Gbps
3D RL Resolution	1°	1°	1°
Reflections	6	6	6
Scenario size (m)	135 × 70 × 18	92.2 × 70 × 15	5.84 × 6.24 × 3.5
Unitary volume analysis	1 m	1 m	0.1 m
Transmitter Position (m)	(78.7, 40.3, 1.3)	(87.8, 32.9, 4)	(3.5, 5.8, 1.05)
